# Beat-to-Beat Blood Pressure Monitoring and Orthostatic Hypotension-Related Falls in Two Cohorts of Older Adults

**DOI:** 10.3390/geriatrics10040102

**Published:** 2025-07-26

**Authors:** Liping Wang, Eveline P. van Poelgeest, Marjolein Klop, Jurgen A. H. R. Claassen, Alfons G. Hoekstra, Nathalie van der Velde

**Affiliations:** 1 Department of Internal Medicine, Geriatrics, Amsterdam University Medical Center, Location University of Amsterdam, Meibergdreef 9, 1105 AZ Amsterdam, The Netherlands; l.wang@amsterdamumc.nl (L.W.); n.vandervelde@amsterdamumc.nl (N.v.d.V.); 2 Amsterdam Public Health, Aging and Later Life, 1081 HV Amsterdam, The Netherlands; 3 Department of Geriatric Medicine, Radboud University Medical Center, 6525 GA Nijmegen, The Netherlandsjurgen.claassen@radboudumc.nl (J.A.H.R.C.); 4 Department of Cardiovascular Sciences, University of Leicester, Leicester LE3 9QP, UK; 5 Computational Science Lab, Informatics Institute, Faculty of Science, University of Amsterdam, 1098 XH Amsterdam, The Netherlands; a.g.hoekstra@uva.nl

**Keywords:** orthostatic hypotension, blood pressure response, falls, older adults, beat-to-beat blood pressure measurements

## Abstract

**Background:** Falls are a major public health issue among older adults, often related to postural or orthostatic hypotension (OH). The optimal timing and methods for measuring blood pressure (BP) to assess OH and its relationship with falls are uncertain. **Methods:** We analyzed data from two older cohorts: the PROHEALTH study (*n* = 30, aged ≥ 65 years) and the NILVAD-CBF trial (*n* = 58, aged ≥ 50 years). Continuous beat-to-beat BP was measured during active stand tests. We assessed orthostatic BP responses during sit-to-stand and supine-to-stand maneuvers and calculated the associations between orthostatic BP response variables and falls. **Results:** In the PROHEALTH cohort, participants with a history of falls exhibited a significantly lower baseline BP (115 ± 13/68 ± 10 vs. 142 ± 21/79 ± 11 mmHg; *p* = 0.004/0.018) and lower systolic BP (SBP) nadir (90 ± 22 vs. 112 ± 25 mmHg; *p* = 0.043) than non-fallers. SBP recovery within three minutes post-stand was delayed in fallers but rapid in non-fallers. A lower resting BP was associated with fall risk, and a lower BP nadir within 10 s after standing showed a trend toward a higher fall risk. No significant associations were found in the NILVAD-CBF cohort (prospective falls). **Conclusions:** Our findings demonstrate that a lower resting SBP and diastolic BP (DBP) are associated with an increased fall risk in older adults, with a lower SBP and DBP nadir after standing also showing a potential association. Persistent OH or delayed BP recovery is identified as a potentially relevant fall risk factor. The supine-to-stand test was more sensitive in detecting OH than the sit-to-stand test. Continuous BP monitoring provides the advantage of detecting pathophysiologic orthostatic BP responses for fall risk assessment in older adults. Further research with larger cohorts is warranted to validate our findings.

## 1. Introduction

Falls represent a significant public health concern, particularly in older adults [1]. The incidence of falls increases with age, especially during the adaptation to postural changes [2]. Orthostatic stresses are common in daily life; an average adult stands up approximately 50 to 60 times per day [3]. The prevalence of postural or orthostatic hypotension (OH) is notably high, affecting approximately 10% of adults aged ≥ 60 years and rising to 30% among those aged ≥ 65 years. Among institutionalized older adults, the prevalence may reach up to 65%, and OH remains common in hospital settings, with rates of approximately 20–30% across inpatient wards [4,5]. OH is associated with an increased risk of falls, fractures, and syncope, particularly in older adults [1,6,7,8,9,10,11]. Given the complexity and medico-legal implications of hospital falls [12], identifying physiological markers such as OH may support improved fall risk assessment across care settings.

Clinically, the active stand test is utilized to diagnose OH and assess pathophysiological compensatory responses to standing. Following a period of sitting or lying down, healthcare providers measure the patient’s blood pressure (BP) and heart rate upon standing. This transition causes a gravity-induced drop in BP within the first 10 s, which is typically countered by baroreflex compensatory mechanisms, such as increased heart rate and peripheral vasoconstriction [13]. The classical definition of OH is a sustained reduction in systolic BP (SBP) of ≥20 mmHg or diastolic BP (DBP) of ≥10 mmHg within three minutes of assuming an upright posture after a supine resting period [14]. Most (inter)national guidelines recommend that the first BP measurements be taken approximately one minute after standing [15,16]. Consequently, the most commonly employed method involves intermittent sphygmomanometer measurements based on the traditional definition of OH.

However, there is ongoing debate regarding the optimal timing for assessing OH and its association with falls [17]. The literature suggests that continuous (beat-to-beat) BP readings may provide a more accurate diagnosis of OH compared with conventional intermittent sphygmomanometer measurements. Various studies indicate that earlier BP measurements (within ≤3 min) after standing may correlate with a higher likelihood of diagnosing unexplained falls. A recent study from The Irish Longitudinal Study on Ageing (TILDA) found that sustained OH occurring 60–120 s after standing was associated with injurious and unexplained falls [18]. Additionally, a systematic review and meta-analysis demonstrated that beat-to-beat BP measurements are more strongly associated with OH and falls compared with intermittent sphygmomanometer measurements [19]. Interestingly, recent observations suggest that in older adults, OH detected between 4 and 6 min may predict fall risk more effectively than OH detected before this time frame, indicating that later measurements may be clinically relevant when OH is suspected as a contributor to falls [1]. For example, a significant initial BP drop (initial OH) is associated with a higher risk of falls, frailty, and syncope in older adults. The prevalence of initial OH is approximately 35% in older outpatients [1,20]. Additionally, evidence suggests that the supine-to-stand measurement has better predictive value for falls, while the sit-to-stand measurement still provides clinically useful insights into orthostatic BP responses and related symptoms [21].

Therefore, the purpose of this study was to characterize BP changes during active standing from a sitting or supine position using continuous (beat-to-beat) BP monitoring across two different cohorts of older individuals. We also aimed to identify which orthostatic BP patterns or OH-related measures best correlate with fall risk.

## 2. Methods

### 2.1. Data Sources

For this explorative study, we analyzed data from two distinct cohorts of older adults from the geriatrics department of Radboud University Medical Center, Nijmegen, the Netherlands. The details of these studies have been published previously [22,23]. In short, the PROHEALTH study was a cross-sectional study in which cerebral oxygenation was measured by near-infrared spectroscopy (NIRS) in combination with continuous BP during postural changes, including 30 participants aged ≥ 65 years, with over 30% recruited from the geriatric outpatient clinic. The NILVAD-cerebral blood flow (NILVAD-CBF) trial was a randomized, placebo-controlled, double-blind trial in individuals with mild-to-moderate Alzheimer’s disease, which included 58 participants aged ≥ 50 years. The key inclusion criteria for NILVAD-CBF were a diagnosis of probable Alzheimer’s disease and a Mini-Mental State Examination (MMSE) score between 12 and 26. In this study, we analyzed baseline data from the NILVAD-CBF trial as an observational cohort, using measurements collected prior to the intervention. The baseline information of the participants included age, sex, body mass index (BMI), current smoking status, history of comorbidities (e.g., cardiovascular disease), and medication use (e.g., antihypertensives). The studies were conducted in accordance with the Declaration of Helsinki and approved by the local Medical Ethical Committee (CMO Arnhem-Nijmegen). Written informed consent was obtained from all participants.

### 2.2. Orthostatic BP Response Assessments

Continuous beat-to-beat BP was assessed through digital finger photoplethysmography (Finapres NOVA, Finapres Medical Systems, Enschede, The Netherlands and Finapres Medical Systems, Amsterdam, The Netherlands) during the active stand tests [24]. In the PROHEALTH study, participants performed supine-to-stand maneuvers and sit-to-stand maneuvers separately. The participants rested for at least 5 min, followed by 3 min of standing [22]. We exclusively analyzed the first maneuvers for this study. For the NILVAD-CBF protocol, participants performed repeated sit-to-stand maneuvers with 2-min sitting intervals and a 5-min standing interval after the last maneuver [23,25]. For this study, we only analyzed the BP measurements during the baseline study visit (before initiating the nilvadipine or placebo treatment intervention), focusing on the BP readings until 5 min of standing. Our OH-related variables of interest were derived from current international guidelines and previously published in scientific publications in the field (Table 1) [14,15,16,20,26].

### 2.3. Falls Outcome

Falls were defined as an unexpected event in which an individual comes to rest on the ground, floor, or a lower level [37]. In the PROHEALTH study, self-reported fall history was collected at baseline and was considered positive if ≥1 fall had occurred in the previous year.

In the NILVAD-CBF trial, fall incidence was assessed prospectively by asking participants about the occurrence of falls during follow-up visits throughout the trial. The study population was categorized dichotomously (positive when ≥1 falls).

### 2.4. Statistical Analysis

The two datasets were analyzed and reported separately in this study due to differences in participant characteristics and heterogeneity in study protocols. Descriptive statistics were applied to summarize the baseline characteristics of the participants. We conducted the Shapiro–Wilk test to decide whether or not our data were normally distributed. For normally distributed variables, we calculated mean values with standard deviations (SDs). For variables that were not normally distributed, we calculated median values with interquartile ranges (IQRs). Categorical data were expressed as frequencies and percentages. The chi-squared test or Fisher’s exact test for used for small samples (categorical data), and the *t*-test or Mann–Whitney U test (continuous normal or non-normal distributed data, respectively) were applied to compare differences between participants who experienced falls and those who did not.

We performed binary logistic regression analysis to calculate odds ratios (ORs) with 95% confidence intervals (CIs) for the quantification of the effect of orthostatic BP responses on falls as an outcome. Given the relatively small sample size, we only adjusted for age and sex as covariates [26], and also adjusted for intervention (nilvadipine or placebo) for the NILVAD-CBF trial.

We analyzed the continuous BP readings by using MATLAB R2022a (Mathworks Inc., Natrick, MA, USA). Signal data that were incomplete or excessively noisy upon visual inspection were excluded from the analysis. Detailed information on the preprocessing approach was published in previous papers [38,39]. Specific OH-related variables (e.g., baseline SBP and DBP; nadir of SBP and DBP) were detected and extracted through custom-written semi-automatic MATLAB scripts [40].

Statistical analyses were carried out using MATLAB and SPSS (version 29.0, IBM Corp, Armonk, NY, USA). We considered *p* < 0.05 as a threshold for statistical significance and *p* < 0.1 as a trend [41,42].

## 3. Results

We studied 88 participants with a mean age of 74 ± 6 years, 30 from the PROHEALTH cohort and 58 from the NILVAD-CBF cohort. In both cohorts, one in four participants had a positive fall history (Table 2).

### 3.1. Sit-to-Stand Maneuver: PROHEALTH Population

**Figure 1:**
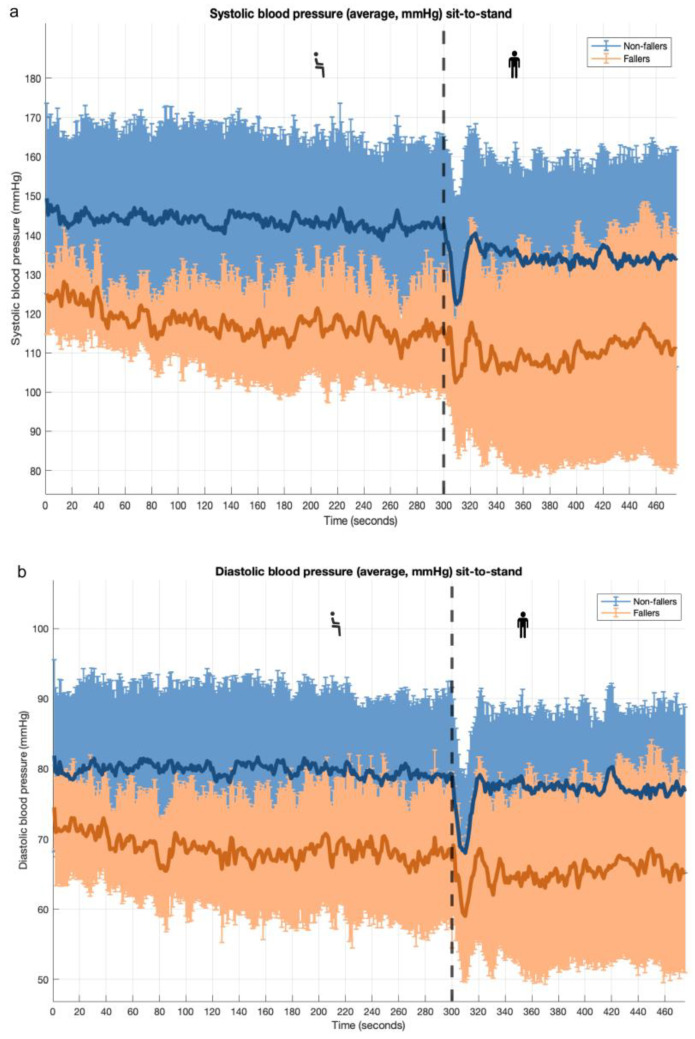
Orthostatic blood pressure response during sit-to-stand maneuver for participants with and without positive falls history (PROHEALTH cohort); mean ± SD. Note: Subfigure (**a**): systolic blood pressure; subfigure (**b**): diastolic blood pressure. Vertical black dashed line indicates moment of standing up.

During the sit-to-stand maneuver, the overall BP response curves were similar between participants with and without a fall history (Figure 1). However, participants with a fall history had a significantly lower resting baseline BP (115 ± 13/68 ± 10 vs. 142 ± 21/79 ± 11 mmHg; *p* = 0.004 and *p* = 0.018, respectively), and a lower SBP nadir (90 ± 22 vs. 112 ± 25 mmHg; *p* = 0.043) compared with those without a fall history (Table 3). A higher resting baseline (systolic, diastolic, and mean arterial) BP was statistically significantly associated with lower odds of having a positive fall history. In addition, there was a trend suggesting that lower orthostatic SBP and DBP nadir values within the first 10 s after standing were associated with higher odds of a positive fall history (Supplementary Table S1).

### 3.2. Supine-to-Stand Maneuver: PROHEALTH Population

**Figure 2:**
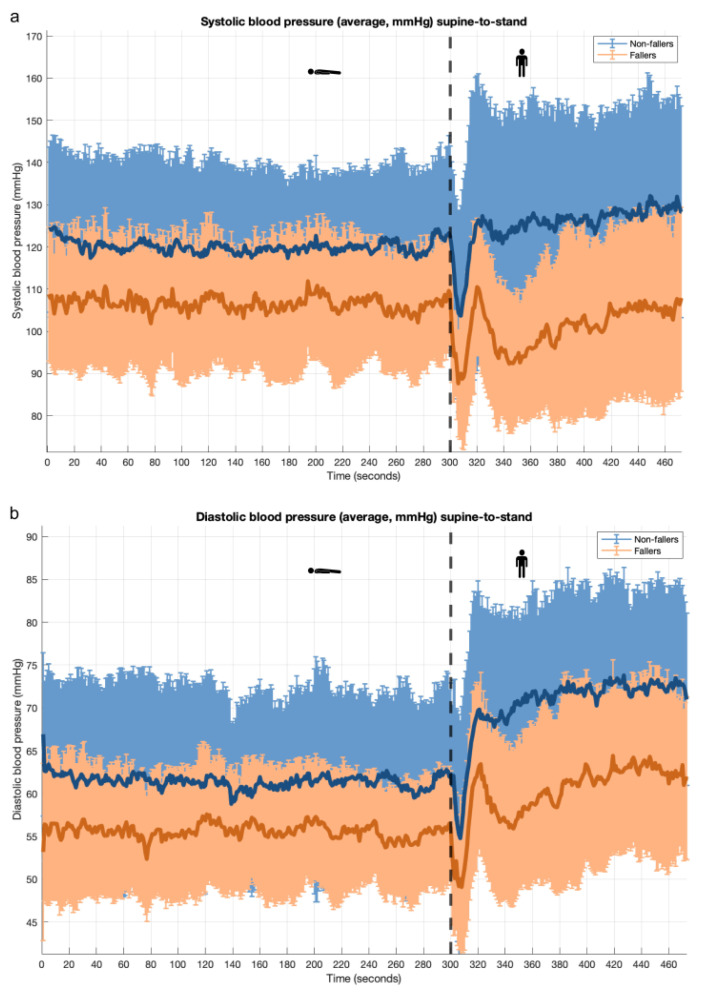
Orthostatic blood pressure response during supine-to-stand maneuver for participants with and without positive fall history (PROHEALTH cohort); mean ± SD. Note: Subfigure (**a**): systolic blood pressure; subfigure (**b**): diastolic blood pressure. Vertical black dashed line indicates moment of standing up.

Similar to the sit-to-stand maneuver, participants with a positive fall history had lower resting supine BP and lower BP nadir values compared with those without a positive fall history, although these differences were not statistically significant (Figure 2 and Supplementary Table S2). In participants without a positive fall history, the SBP returned to normal or exceeded baseline levels shortly after standing, whereas in participants with a fall history, the SBP did not return to baseline levels within 3 min (Figure 2).

### 3.3. Sit-to-Stand Maneuver: NILVAD-CBF Population

No significant associations were found between fallers and non-fallers during follow-up and baseline BP, nor between falls and various BP responses/variables (Supplementary Figure S1 and Supplementary Table S3).

## 4. Discussion

We observed significant differences in baseline resting sitting BP and SBP nadir values between participants with and without a history of falls during the sit-to-stand maneuver in the PROHEALTH cohort. A similar pattern was seen during the supine-to-stand maneuver, although the differences were not statistically significant. We also observed that, during the supine-to-stand maneuver, recovery to baseline BP was not achieved after 3 min in fallers, in contrast with non-fallers. In the sit-to-stand maneuver, a lower resting BP was associated with fall risk, and a lower BP nadir within 10 s after standing showed a trend toward a higher fall risk. No significant associations were found between other orthostatic BP responses identified in the literature and fall risk in our cohort.

Our finding that the baseline resting BP significantly differed between participants with and without a history of falls is consistent with previous reports indicating that a lower BP is associated with an increased fall risk in older adults. For example, a study among the oldest old (≥85 years) suggested a relationship between low BP and fall risk [43]. Additionally, a prospective study found that fallers had significantly lower resting supine BP and lower BP nadirs after standing, which aligns with our results [37]. However, evidence from the literature on the relationship between BP values and falls in older individuals remains inconclusive [19]. This variability may be attributed to differences in risk based on specific patient characteristics. For instance, in Swedish older adults (≥60 years), the relationship between BP and fall risk varied by functional status: in individuals with functional impairment, low BP was associated with an increased probability of falls, whereas in those without functional impairment, high BP correlated with a higher fall risk [44]. Additionally, baseline BP can modulate the severity of OH and its clinical impact, especially in individuals with hypertension or impaired BP recovery [45]. However, a meta-analysis of 63 studies showed that adjusting for baseline BP does not affect the association between OH and falls [10]. Moreover, baseline resting BP values were lower in fallers from the PROHEALTH population and, thus, possibly in need of deprescribing antihypertensives.

Our observation of differing SBP recovery patterns between fallers and non-fallers, specifically the delayed recovery to baseline values in fallers during the supine-to-stand maneuvers, may have significant clinical implications. Based on the data from TILDA, a large observational study of community-dwelling individuals, orthostatic BP values are expected to stabilize within 60 s for males aged ≥ 70 years and within 90 s for females aged ≥ 80 years (comparable to our study population) [26]. Fallers in our analysis exhibited marked delays in BP recovery compared with these reference values immediately after standing. This phenomenon has been previously observed in a small subgroup of older TILDA participants who appeared at increased risk for unexplained and injurious falls. They demonstrated slow BP recovery, particularly in individuals with significant initial BP drops [26,28]. Additionally, older adults in long-term care facilities who fell in the previous year exhibited more pronounced SBP drops and significantly delayed SBP recovery compared with non-fallers [46].

Continuous BP measurement allows for the detection of delayed BP recovery, defined as an SBP drop of >20 mmHg and/or a DBP drop of ≥10 mmHg at 30–40 s, 60 s, and 90 s after standing [17,26,30,34,35], and sustained OH, defined as an SBP drop of ≥20 mmHg from 60 to 180 s after standing [15,26,29,30]. Accurately diagnosing this delayed BP recovery appears to be crucial for evaluating (unexplained) fall risk in older adults [16,17,26,29,30,35]. Moreover, observational studies indicate that delayed BP recovery is associated with other adverse health outcomes in older adults, including all-cause mortality, cognitive decline, incident dementia, and functional decline [3]. This clinically relevant diagnosis cannot be made using traditional sphygmomanometers or semi-automatic electronic devices, which typically take 15 to 45 s per measurement and require at least a 1-min interval between measurements to allow for arm blood flow recovery [30].

Furthermore, our findings underscore the clinical relevance of continuous BP monitoring, which can detect transient orthostatic BP responses that may be missed by conventional intermittent measures. Notably, the supine-to-stand test proved more sensitive than the sit-to-stand test in identifying OH-related responses. Our findings suggest that the supine-to-stand maneuver detected more cases of OH (and its variants) compared with the sit-to-stand maneuver, consistent with previous reports [21,47]. This difference is likely due to the greater magnitude of the postural change and increased gravitational stress upon standing from a supine position [21]. A systematic review and meta-analysis demonstrated that both classical OH and systolic OH were associated with falls [10]. Similarly, a recent large study reported that supine systolic OH was linked to an increased fall risk [21]. In line with these findings, we observed delayed recovery of supine SBP after standing. However, in our relatively small sample, we did not observe a significant association between different types of OH and fall risk in either maneuver. Larger studies are needed to further investigate the associations between OH (and its variants) and fall risk using both supine-to-stand and sit-to-stand tests. Supine-to-stand testing with continuous BP monitoring is recommended in the World Falls Guidelines when OH is suspected but not confirmed by traditional tools [15] and may support more accurate fall risk assessment in geriatric care.

The differences in findings between the PROHEALTH and NILVAD-CBF cohorts may reflect underlying differences in participant characteristics. PROHEALTH included cognitively preserved older adults, while NILVAD-CBF participants with mild-to-moderate Alzheimer’s disease may exhibit altered autonomic and cardiovascular regulation. Additionally, medication use and comorbidity burden were likely higher in the NILVAD-CBF cohort, potentially influencing orthostatic BP responses and fall risk. These factors may have contributed to the absence of significant associations in NILVAD-CBF, highlighting the importance of considering population-specific characteristics when interpreting orthostatic response patterns and fall risk.

Our explorative study had several limitations. First, our sample size was relatively small, with a low prevalence of OH (particularly during sit-to-stand maneuvers) and falls, which may have limited our ability to detect associations with falls. Additionally, we could only control for a limited number of covariates (age, sex, and intervention) and did not account for other established fall risk factors, such as fall risk-increasing drugs (FRIDs), cognitive status, pharmacologic burden, and autonomic function. Nonetheless, the distinct BP changes and recovery patterns observed in this small sample of older community-dwelling individuals may indicate clinically meaningful differences in orthostatic BP responses contributing to fall risk. We analyzed the two cohorts separately due to differences in participant characteristics, fall classification (retrospective vs. prospective), and orthostatic testing protocols. However, this procedural heterogeneity may limit internal consistency and should be considered when interpreting findings. Retrospective falls outcome classification may have led to misclassification due to recall bias [48,49]. In the NILVAD study, baseline active stand tests were performed prior to randomization, while fall outcomes were assessed prospectively during the study follow-up. However, fall dates were reported retrospectively at predefined follow-up visits (e.g., at weeks 6, 26, 39, 52, and 78) and were occasionally incomplete or inconsistent with the expected follow-up period. Therefore, we were not able to perform exact time-to-event analyses. Although intervention effects (nilvadipine vs. placebo) may have influenced BP responses, this potential influence was likely limited, as the intervention was adjusted for in the analysis. Furthermore, nilvadipine, a dihydropyridine calcium channel blocker, has a relatively low risk of inducing OH compared with other antihypertensives [50,51,52], as supported by findings from the large NILVAD trial [50].

Since the cohorts differed in participant characteristics and measurement protocols, analyzing them separately allowed us to provide complementary insights into the association between different orthostatic BP responses and fall risk across distinct older populations. With this approach, a broader understanding of these physiological patterns was obtained, pointing toward the need for future studies to develop more tailored orthostatic hypotension assessments for diverse older populations. Despite its limited sample size and inability to adjust for all relevant confounders, including cognitive status, comorbidities, autonomic function, and FRIDs, this study contributes valuable insights to the complex hemodynamic profiles linked to fall risk in the geriatric population, suggesting that individualized fall risk assessment could benefit from more precise and dynamic BP-monitoring techniques.

Future research involving larger, longitudinal datasets and additional physiological parameters is warranted to refine these associations and inform targeted interventions. These findings highlight the importance of incorporating integrated, evidence-based strategies into the clinical management of fall risk in older adults [15,53]. Moreover, advanced analytical approaches, such as function-on-scalar regression models [54], could be applied to assess hemodynamic parameters (e.g., heart rate dynamics) during postural transitions, providing additional insights into autonomic regulation and fall risk.

## 5. Conclusions

In this project, using data from two distinct cohorts of older adults, we observed that lower resting SBP and DBP values are associated with an increased fall risk in older adults, with lower SBP and DBP nadir values after standing also showing a potential association. Furthermore, in this small explorative study, we also observed a trend toward delayed BP recovery, or sustained OH, being present in fallers but not in non-fallers after supine-to-standing maneuvers. The supine-to-stand test was more sensitive in detecting OH than the sit-to-stand test. Our findings reinforce previous research showing that beat-to-beat (continuous) BP measurements enable a more advanced analysis of orthostatic BP responses during postural changes relevant in fall risk assessment. Specifically, we identified key pathophysiological impairments in postural BP control, which are significant risk factors for falls, syncope, and related morbidity and costs in the older population. Early identification of these orthostatic BP responses associated with fall risk allows for timely intervention and management, potentially reducing fall-related morbidity and mortality in older adults. However, larger cohorts and prospective longitudinal studies are necessary to validate and expand upon our findings.

## Figures and Tables

**Table 1:** Definitions, units, abbreviations, and calculation of variables used and orthostatic symptoms.

Variable	Description
Baseline (resting) supine/sitting SBP	Mean SBP value of 30 s–60 s before standing, as baseline [26]; mmHg
Baseline (resting) supine/sitting DBP	Mean DBP value of 30 s–60 s before standing, as baseline [26]; mmHg
Baseline (resting) supine/sitting MAP	Mean MAP value of 30 s–60 s before standing, as baseline; MAP = DBP + 1/3 × (SBP–DBP) [27] (which is the same as ‘mean SBP’ + 1/3 × (‘mean SBP’–‘mean DBP’); mmHg
Orthostatic BP responses, variants, and symptoms	
SBP nadir	Minimum SBP value during standing; mmHg
DBP nadir	Minimum DBP value during standing; mmHg
MAP nadir	Minimum MAP value during standing; mmHg
Time to SBP nadir	Related time to SBP nadir during standing; seconds
Time to DBP nadir	Related time to DBP nadir during standing; seconds
Time to MAP nadir	Related time to MAP nadir during standing; seconds
Change in SBP from baseline	SBP(t) on standing–baseline (resting) supine/sitting SBP; ΔSBP(t); mmHg
Change in DBP from baseline	DBP(t) on standing–baseline (resting) supine/sitting DBP; ΔDBP(t); mmHg
Change in MAP from baseline	MAP(t) on standing–baseline (resting) supine/sitting MAP; ΔMAP(t); mmHg
Largest drop in SBP	Maximum change in SBP from baseline, SBP nadir–baseline (resting) supine/sitting SBP; ΔSBPmax; mmHg [22]
Largest drop in DBP	Maximum change in DBP from baseline, DBP nadir–baseline (resting) supine/sitting DBP; ΔDBPmax; mmHg [22]
Largest drop in MAP	Maximum change in MAP from baseline, MAP nadir–baseline (resting) supine/sitting MAP; ΔMAPmax; mmHg
Initial OH (15 s)	A transient BP decrease that exceeds thresholds (a drop in SBP of ≥40 mmHg and/or DBP of ≥20 mmHg) within 15 s of standing [16,26]
Sustained OH (10 s interval: 60–110 s)	A sustained SBP drop of ≥20 mmHg or a DBP drop of ≥10 mmHg upon standing, defined as exceeding the thresholds at all following time points: 60, 70, 80, 90, 100, and 110 s [26,28]
Sustained OH (1 min interval: 1–3 mins)	A sustained SBP drop of ≥20 mmHg or a DBP drop of ≥10 mmHg upon standing, defined as exceeding the thresholds at least 2 of the following time points: 1, 2, and 3 min. A sustained decline in SBP of ≥20 mmHg occurring 60–180 s after standing [15,16,19,29,30]
Classical OH (3 mins)	A sustained SBP drop of ≥20 mmHg and/or a DBP drop of ≥10 mmHg upon standing, defined as exceeding these thresholds at all following time points: 1, 2, and 3 min [10,31]
Delayed OH (after 3 mins)	A drop that exceeded the thresholds (an SBP drop of ≥20 mmHg or a DBP drop of ≥10 mmHg upon standing) at the following time points: 4 or 5 min [10,15,31]
Orthostatic BP recovery	Full recovery: >95% recovery of SBP/DBP at 60 s relative to baseline; Partial recovery: 80–95% recovery of SBP/DBP at 60 s relative to baseline; No recovery: <80% recovery of SBP/DBP at 60 s relative to baseline [32,33]
Delayed orthostatic BP recovery	An SBP drop of >20 mmHg and/or a DBP drop of ≥10 mmHg at 30–40 s, 60 s, and 90 s after standing, without meeting the criteria of classical OH [17,26,30,34,35]
Orthostatic intolerance	Symptoms of OH or complaints of participants (e.g., feeling dizzy, lightheadedness, or feeling unstable) during OH measurements [36]

Notes: BP, blood pressure; DBP, diastolic blood pressure; MAP, mean arterial pressure; mins, minutes; OH, orthostatic hypotension; SBP, systolic blood pressure; s, second.

**Table 2:** Baseline characteristics of participants.

	PROHEALTH	NILVAD-CBF	Total
Number of participants (*n*)	30	58	88
Age (years; mean ±SD)	74 ± 7	73 ± 6	74 ± 6
Female sex (*n*; %)	11 (37)	34 (59)	45 (51)
BMI (kg/m^2^; mean ±SD)	24 ± 3	25 ± 4	24 ± 3
Currently smoking (*n*; %)	1 (3)	-	1 (3)
MMSE score (median, IQR)	-	21 (12–26)	21 (12–26)
MoCA score (median, IQR)	26 (24–28)	-	26 (24–28)
DAD score (median, IQR)	-	34 (30–38)	34 (30–38)
Cardiovascular disease (*n*; %)	4 (13)	9 (16)	13 (15)
Diabetes mellitus (*n*; %)	3 (10)	3 (5)	6 (7)
Depression (*n*; %)	3 (10)	-	3 (10)
Antihypertensive drug use (*n*; %)	7 (23)	17 (29)	24 (27)
Antidepressant use (*n*; %)	2 (7)	8 (14)	10 (11)
Cholinesterase inhibitor use (*n*; %)	-	46 (79)	46 (52)
Statin use (*n*; %)	3 (10)	10 (17)	13 (15)
Falls (*n*; %)	7 (23)	14 (24)	21(24)

Notes: BMI, body mass index; DAD, Disability Assessment for Dementia (maximum score = 100); IQR, interquartile range; MMSE, Mini-Mental State Examination (maximum score = 30); MoCA, Montreal Cognitive Assessment (maximum score = 30); SD, standard deviation.

**Table 3:** Comparison of orthostatic blood pressure responses and orthostatic intolerance for participants with and without positive fall history (sit-to-stand maneuver; PROHEALTH cohort).

Variable	All	Fall Previous Year	No Falls Previous Year	*p*
Number of participants	30	7	23	
Baseline (resting) sitting SBP (mmHg; mean ± SD)	136 (22)	115 (13)	142 (21)	0.004 *
Baseline (resting) sitting DBP (mmHg; mean ± SD)	77 (12)	68 (10)	79 (11)	0.018 *
Baseline (resting) sitting MAP (mmHg; mean ± SD)	96 (14)	84 (11)	100 (13)	0.004 *
SBP nadir (mmHg; mean ± SD)	107 (26)	90 (22)	112 (25)	0.043 *
DBP nadir (mmHg; mean ± SD)	60 (11)	53 (10)	62 (11)	**0.059**
MAP nadir (mmHg; mean ± SD)	72 (17)	65 (14)	74 (18)	0.234
Time to SBP nadir (seconds; median, IQR)	10 (8–86)	9 (8–34)	10 (8–88)	0.573
Time to DBP nadir (seconds; median, IQR)	8 (6–31)	8 (6–8)	9 (6–38)	0.364
Time to MAP nadir (seconds; median, IQR)	22 (8–54)	8 (7–16)	26 (8–54)	0.239
Largest drop in SBP (mmHg; mean ± SD)	−29 (14)	−26 (13)	−30 (14)	0.435
Largest drop in DBP (mmHg; mean ± SD)	−16 (6)	−15 (3)	−17 (6)	0.332
Largest drop in MAP (mmHg; mean ± SD)	−21 (8)	−18 (6)	−21 (8)	0.340
Initial OH (at 15 s), *n* (%)	1 (3)	0 (0)	1 (4)	1.000
Sustained OH (60–110 s), *n* (%)	2 (7)	1 (14)	1 (4)	0.418
Sustained OH (1–3 min), *n* (%)	5 (17)	2 (29)	3 (13)	0.565
Classical OH (3 min), *n* (%)	2 (7)	1 (14)	1 (4)	0.418
Orthostatic BP full recovery at 60 s, *n* (%)	19 (63)	4 (57)	15 (65)	0.182
Orthostatic BP partial recovery at 60 s, *n* (%)	10 (33)	2 (29)	8 (35)	
Orthostatic BP no recovery at 60 s, *n* (%)	1 (3)	1 (14)	0 (0)	
Delayed orthostatic BP recovery, *n* (%)	12 (40)	3 (43)	9 (39)	0.894
Orthostatic intolerance during stand, *n* (%)	1 (3)	1 (14)	0 (0)	0.233

Notes: BP, blood pressure; DBP, diastolic blood pressure; IQR, interquartile range; MAP, mean arterial pressure; mins, minutes; OH, orthostatic hypotension; SBP, systolic blood pressure; s, second; SD, standard deviation; statistically significant differences (*p* < 0.05) are in bold with *; a trend is in bold.

## Data Availability

In compliance with European data protection laws and the conditions of the trial’s ethical approval, we are unable to disclose any patients’ personal data, even if anonymized. Researchers interested in access to the trial data may, upon request, apply for access.

## Supplementary Materials

The following supporting information can be downloaded at https://www.mdpi.com/article/10.3390/geriatrics10040102/s1. Figure S1: Orthostatic blood pressure responses to active standing of participants who fell and who did not fall during follow-up (sit-to-stand maneuver; NILVAD-CBF trial); Table S1: Association between orthostatic blood pressure response and falls history (sit-to-stand maneuver; PROHEALTH cohort); Table S2: Comparison of orthostatic blood pressure responses and orthostatic intolerance for participants with and without positive fall history (supine-to-stand maneuver; PROHEALTH cohort); Table S3: Comparison of orthostatic blood pressure responses and orthostatic intolerance for participants who fell and those who did not fall during follow-up (sit-to-stand maneuver; NILVAD-CBF trial).
