# Supplementary material for: Beat-to-Beat Blood Pressure Monitoring and Orthostatic Hypotension-Related Falls in Two Cohorts of Older Adults
Source: Geriatrics (Basel). 2025 Jul 26;10(4):102. doi: 10.3390/geriatrics10040102 (PMC12385785; doi:10.3390/geriatrics10040102)
Supplement: Supplementary file 1 [file geriatrics-10-00102-s001.zip › geriatrics-3640303-supplementary.pdf]

Supplementary tables and figures

Beat-to-beat blood pressure monitoring and orthostatic hypotension-related falls in two cohorts of older adults

Liping Wang, Eveline P. van Poelgeest*, Marjolein Klop, Jurgen A.H.R. Claassen, Alfons G. Hoekstra, Nathalie van der Velde

*Corresponding author:

Eveline P. van Poelgeest, MD PhD, internist-geriatrician and clinical pharmacologist

Amsterdam University Medical Centers, Location University Medical Center,

Department of Internal Medicine/Geriatrics, Meibergdreef 9, Amsterdam, The Netherlands

Email: e.p.vanpoelgeest@amsterdamumc.nl

Supplementary Table S1 Association between orthostatic blood pressure response and falls history (sit-to-stand maneuver; PROHEALTH cohort)

	**Unadjusted**			**Adjusted**		
**Variable**	**OR**	**95% CI**	***P***	**OR**	**95% CI**	***P***
Baseline (resting) sitting SBP	0.91	0.84-0.98	**0.016***	0.86	0.74-0.99	**0.044***
Baseline (resting) sitting DBP	0.88	0.78-0.99	**0.040***	0.88	0.78-0.99	**0.049***
Baseline (resting) sitting MAP	0.86	0.75-0.98	**0.023***	0.83	0.70-0.99	**0.039***
SBP nadir	0.96	0.91-1.01	**0.064**	0.93	0.86-1.00	**0.061**
DBP nadir	0.91	0.81-1.01	**0.086**	0.90	0.81-1.01	**0.083**
MAP nadir	0.97	0.93-1.02	0.248	0.98	0.93-1.04	0.455
Time to SBP nadir	0.99	0.98-1.01	0.561	0.99	0.98-1.01	0.567
Time to DBP nadir	0.98	0.95-1.02	0.348	0.98	0.95-1.02	0.296
Time to MAP nadir	0.98	0.95-1.02	0.273	0.98	0.95-1.01	0.234
Largest drop in SBP	1.03	0.96-1.10	0.424	1.02	0.95-1.10	0.537
Largest drop in DBP	1.09	0.91-1.31	0.232	1.07	0.88-1.30	0.507
Largest drop in MAP	1.07	0.93-1.21	0.331	1.05	0.92-1.21	0.480
Sustained OH (60-110s)	3.67	0.20-67.65	0.382	4.34	0.20-93.12	0.348
Sustained OH (1-3mins)	2.67	0.35-20.51	0.346	3.12	0.38-25.71	0.291
Classical OH (3mins)	3.67	0.20-67.65	0.382	4.34	0.20-93.12	0.348
Orthostatic BP recovery (partial)	0.94	0.14-6.28	0.947	1.29	0.17-9.76	0.808

Notes: The model was adjusted for age and sex, considering the small sample size of the study. Initial OH, time to BP partially recovered, and orthostatic intolerance during stand were not reported due to very low number of events observed for these variables, which was not feasible to calculate meaningful ORs. BP, blood pressure; CI, confidence interval; DBP, diastolic blood pressure; MAP, mean arterial pressure; mins, minutes; OH, orthostatic hypotension; OR, odds ratio; SBP, systolic blood pressure; s, second; statistically significant differences ($p < 0.05$) are in bold with *; a trend is in bold.

Supplementary Table S2 Comparison of orthostatic blood pressure responses and orthostatic intolerance for participants with and without positive fall history (supine-to-stand maneuver; PROHEALTH cohort)

**Variable**	**All**	**Fall previous year**	**No fall previous year**	***P***
Number of participants	30	7	23	
Baseline (resting) supine SBP, mmHg, mean (SD)	117 (20)	107 (20)	120 (19)	0.108
Baseline (resting) supine DBP, mmHg, mean (SD)	60 (10)	54.5 (7)	61 (10)	0.114
Baseline (resting) supine MAP, mmHg, mean (SD)	79 (12)	71.9 (11)	81 (11)	**0.073**
SBP nadir, mmHg, mean (SD)	87 (25)	74 (18)	91 (25)	0.109
DBP nadir, mmHg, mean (SD)	47 (12)	43 (8)	49 (13)	0.257
MAP nadir, mmHg, mean (SD)	61 (15)	53 (11)	63 (16)	0.140
Time to SBP nadir, s, median (IQR)	7 (5-9)	6 (3-31)	7 (5-9)	0.864
Time to DBP nadir, s, median (IQR)	6 (4-10)	5 (2-73)	6 (4-9)	0.844
Time to MAP nadir, s, median (IQR)	6 (4-12)	6 (3-59)	6 (5-10)	0.750
Largest drop in SBP, mmHg, mean (SD)	-30 (21)	-33 (29)	-29 (19)	0.704
Largest drop in DBP, mmHg, mean (SD)	-12 (10)	-12 (9)	-13 (11)	0.872
Largest drop in MAP, mmHg, mean (SD)	-18 (13)	-19 (16)	-18 (13)	0.870
Initial OH (15s), n (%)	9 (30)	1 (14)	8 (35)	0.393
Sustained OH (60-110s), n (%)	10 (33)	3 (43)	7 (30)	0.657
Sustained OH (1-3mins), n (%)	16 (53)	4 (57)	12 (52)	1.000
Classical OH (3mins), n (%)	6 (20)	2 (29)	4 (17)	0.603
Orthostatic BP full recovery at 60s, n (%)	26 (87)	6 (86)	20 (87)	
Orthostatic BP partial recovery at 60s, n (%)	3 (10)	1 (14)	2 (9)	0.789
Orthostatic BP no recovery at 60s, n (%)	1 (3)	0 (0)	1 (4)	
Delayed orthostatic BP recovery, n (%)	5 (40)	2 (29)	3 (13)	0.628
Orthostatic intolerance during stand, n (%)	5 (13)	2 (29)	3 (13)	0.565

Notes: BP, blood pressure; DBP, diastolic blood pressure; IQR, interquartile Range; MAP, mean arterial pressure; mins, minutes; OH, orthostatic hypotension; SBP, systolic blood pressure; s, second; SD, standard deviation; statistically significant differences (p<0.05) are in bold with *, a trend is in bold.

Supplementary Figure S1 Orthostatic blood pressure responses to active standing of participants who fell and who did not fall during follow-up (sit-to-stand maneuver; NILVAD-CBF trial), mean±SD

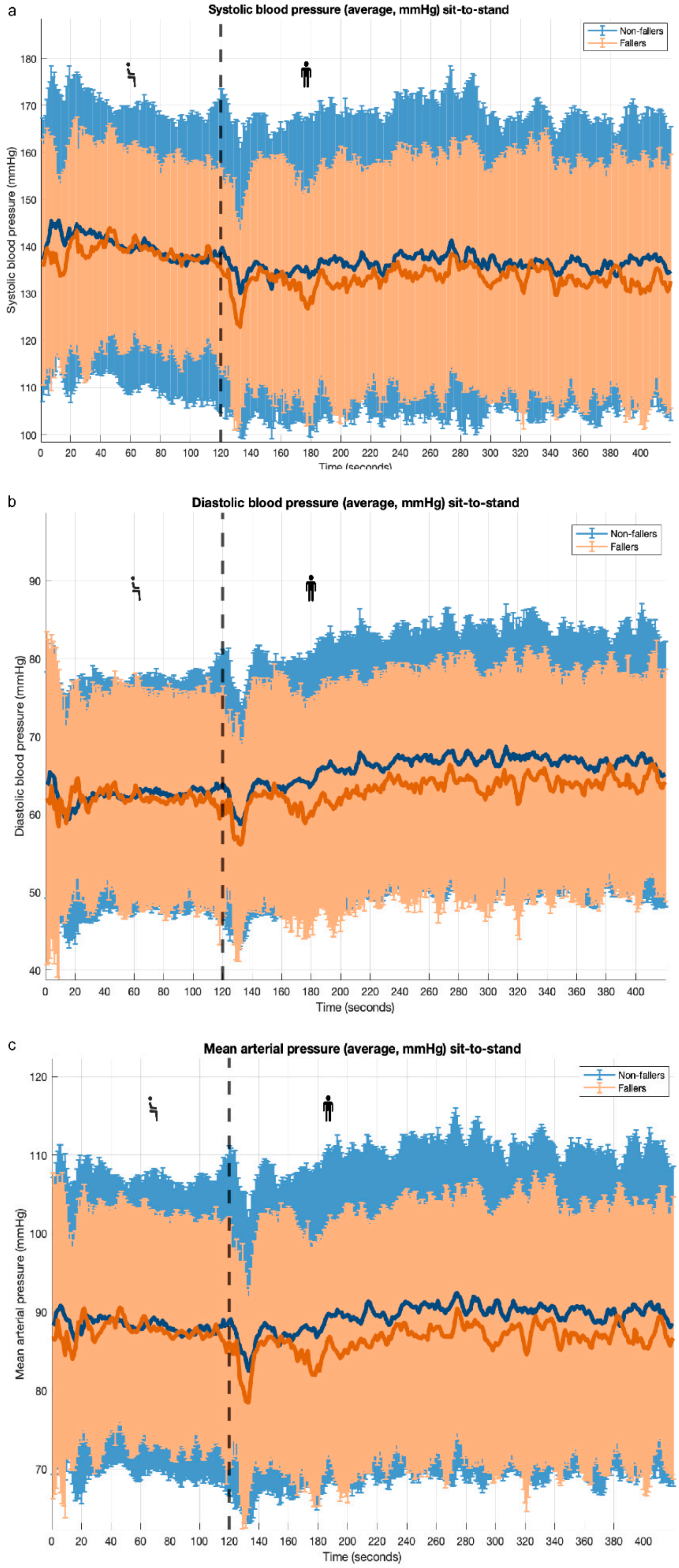

Note: Subfigure a: systolic blood pressure; subfigure b: diastolic blood pressure; subfigure c: mean arterial pressure. Vertical black dashed line indicates moment of standing up.

Supplementary Table S3 Comparison of orthostatic blood pressure responses and orthostatic intolerance for participants who fell and those who did not fall during follow-up (sit-to-stand maneuver; NILVAD-CBF trial)

**Variable**	**All**	**Fall during study follow-up**	**No fall during study follow-up**	***P***
Number of participants	55	13	42	
Baseline (resting) sitting SBP, mmHg, mean (SD)	136 (29)	134 (23)	136 (31)	0.802
Baseline (resting) sitting DBP, mmHg, mean (SD)	65 (16)	63 (14)	65 (16)	0.661
Baseline (resting) sitting MAP, mmHg, mean (SD)	88 (19)	87 (16)	89 (20)	0.716
SBP nadir, mmHg, mean (SD)	109 (28)	107 (23)	110 (30)	0.760
DBP nadir, mmHg, mean (SD)	50 (15)	49 (13)	51 (15)	0.778
MAP nadir, mmHg, mean (SD)	69 (20)	69 (15)	68 (21)	0.985
Time to SBP nadir, s, median (IQR)	13 (10-82)	13 (9-16)	13 (11-108)	0.417
Time to DBP nadir, s, median (IQR)	10 (7-127)	10 (7-34)	10 (7-172)	0.677
Time to MAP nadir, s, median (IQR)	11 (8-119)	10 (9-28)	11 (8-126)	0.670
Largest drop in SBP, mmHg, mean (SD)	-26 (11)	-27 (6)	-26 (13)	0.867
Largest drop in DBP, mmHg, mean (SD)	-14 (5)	-14 (4)	-15 (6)	0.607
Largest drop in MAP, mmHg, mean (SD)	-18 (6)	-18 (4)	-19 (7)	0.832
Initial OH (15s), n (%)	1 (2)	0 (0)	1 (3)	1.000
Sustained OH (1-3mins), n (%)	2 (4)	0 (0)	2 (5)	1.000
Classical OH (3mins), n (%)	1 (2)	0 (0)	1 (2)	1.000
Delayed OH after 3mins of standing, n (%)	8 (15)	1 (8)	7 (17)	0.664
Orthostatic BP full recovery at 60s, n (%)	44 (80)	10 (77)	34 (81)	0.709
Orthostatic BP partial recovery at 60s, n (%)	11 (20)	3 (23)	8 (19)	
Delayed orthostatic BP recovery, n (%)	12 (22)	3 (23)	9 (21)	0.723
Orthostatic intolerance during stand, n (%)	3 (6)	1 (8)	2 (5)	1.000

Notes: BP, blood pressure; DBP, diastolic blood pressure; IQR, interquartile Range; MAP, mean arterial pressure; mins, minutes; OH, orthostatic hypotension; SBP, systolic blood pressure; s, second; SD, standard deviation.
